# Supplementary material for: Bullying and Health Related Quality of Life among Adolescents—A Systematic Review
Source: Children (Basel). 2022 May 24;9(6):766. doi: 10.3390/children9060766 (PMC9222044; doi:10.3390/children9060766)
Supplement: Supplementary file 1 [file children-09-00766-s001.zip › Supplementary File S1.pdf]

Newcastle-Ottawa Scale adapted for cross-sectional studies

Selection: (Maximum 5 stars)

1) Representativeness of the sample:
   a) Truly representative of the average in the target population. * (all subjects or random sampling)
   b) Somewhat representative of the average in the target population. * (non-random sampling)
   c) Selected group of users.
   d) No description of the sampling strategy.

2) Sample size:
   a) Justified and satisfactory. *
   b) Not justified.

3) Non-respondents:
   a) Comparability between respondents and non-respondents characteristics is established, and the response rate is satisfactory. *
   b) The response rate is unsatisfactory, or the comparability between respondents and non-respondents is unsatisfactory.
   c) No description of the response rate or the characteristics of the responders and the non-responders.

4) Ascertainment of the exposure (risk factor):
   a) Validated measurement tool. **
   b) Non-validated measurement tool, but the tool is available or described.*
   c) No description of the measurement tool.

Comparability: (Maximum 2 stars)

1) The subjects in different outcome groups are comparable, based on the study design or analysis. Confounding factors are controlled.
   a) The study controls for the most important factor (select one). *
   b) The study control for any additional factor. *

Outcome: (Maximum 3 stars)

1) Assessment of the outcome:
   a) Independent blind assessment. **
   b) Record linkage. **
   c) Self report. *
   d) No description.

2) Statistical test:
   a) The statistical test used to analyze the data is clearly described and appropriate, and the measurement of the association is presented, including confidence intervals and the probability level (p value). *
   b) The statistical test is not appropriate, not described or incomplete.

This scale has been adapted from the Newcastle-Ottawa Quality Assessment Scale for cohort studies to perform a quality assessment of cross-sectional studies for the systematic review, "**Bullying and health related quality of life among adolescents- a systematic review**".
